# A nomogram combining neutrophil to lymphocyte ratio (NLR) and prognostic nutritional index (PNI) to predict distant metastasis in gastric cancer

**DOI:** 10.1038/s41598-024-65307-7

**Published:** 2024-07-04

**Authors:** Jiawei Liu, Ruizheng Sun, Kaimei Cai, Yi Xu, Weijie Yuan

**Affiliations:** 1 Department of Gastrointestinal Surgery, Xiangya Hospital, Central South University (CSU), Changsha, Hunan China; 2 The Hunan Provincial Key Lab of Precision Diagnosis and Treatment for Gastrointestinal Tumor, Xiangya Hospital, Central South University (CSU), Changsha, Hunan China; 3 National Clinical Research Center for Geriatric Disorders, Xiangya Hospital, Central South University (CSU), Changsha, Hunan China

**Keywords:** Gastric cancer, Distant metastasis, Nomogram, Blood parameters, Cancer, Gastrointestinal cancer, Metastasis, Tumour biomarkers

## Abstract

In this study, We aim to explore the association between the neutrophil to lymphocyte ratio (NLR), platelet to lymphocyte ratio (PLR), systemic immune-inflammatory index (SII), lymphocyte to monocyte ratio (LMR) and prognostic nutritional index (PNI) and distant metastasis of gastric cancer and develop an efficient nomogram for screening patients with distant metastasis. A total of 1281 inpatients with gastric cancer were enrolled and divided into the training and validation set.Univariate, Lasso regression and Multivariate Logistic Regression Analysis was used to identify the risk factors of distant metastasis. The independent predictive factors were then enrolled in the nomogram model. The nomogram’s predictive perform and clinical practicality was evaluated by receiver operating characteristics (ROC) curves, calibration curves and decision curve analysis. Multivariate Logistic Regression Analysis identified d-dimer, CA199, CA125, NLR and PNI as independent predictive factors. The area under the curve of our nomogram based on these factors was 0.838 in the training cohort and 0.811 in the validation cohort. The calibration plots and decision curves demonstrated the nomogram’s good predictive performance and clinical practicality in both training and validation cohort. Therefore,our nomogram could be an important tool for clinicians in screening gastric cancer patients with distant metastasis.

## Introduction

Gastric cancer (GC) is a global health problem, with more than 1 million new cases each year. Despite the incidence and mortality have decreased over the past 50 years, the mortality of stomach cancer remains the fourth rank worldwide^1^. Although the 5-year survival rate for gastric cancer is gradually increasing, the prognosis of patients with distant metastasis is still unsatisfactory^2,3^. Patients with distant metastatic spread are recommended to accept comprehensive treatment based on systemic antitumor therapy, which may help to prolong survival and improve the quality of life^4–6^. Accurate screening of patients with distant metastasis is crucial for providing optimal treatments and avoiding unnecessary surgery operation.

It has been widely recognized that inflammation is one of the hallmarks of cancer and plays decisive roles in the initiation, development invasion, and metastasis of tumors^7–10^. Since Rudolf Virchow’s initial discovery of a connection between inflammation and cancer in 1863, an increasing number of studies have demonstrated the roles of local immune response and systemic inflammation in tumorigenesis and progression of tumors. Epidemiologic studies have shown that 2.2 million new cancer cases were attributable to infections, representing 13% of all cancer cases (excluding non-melanoma skin cancers)^11^. Some systemic inflammation markers based on peripheral blood indicators such as neutrophil to lymphocyte ratio (NLR), platelet to lymphocyte ratio (PLR), systemic immune-inflammatory index (SII) and lymphocyte to monocyte ratio (LMR) have been confirmed to be potential predictors of multiple malignant tumors^12–19^.

Compared to patients with no metastasis, metastatic patients had poorer nutritional status^20^. Prognostic nutritional index (PNI), which reflects the nutritional statuses of patients, has been referred to as a prognostic factor in multiple malignancies^21–26^.

Although the association between the prognosis of gastric cancer and composite inflammatory and nutritional markers has been recognized, the value of them in diagnosing distant metastasis in gastric cancer still requires exploration. In this study, we aim to explore the association between NLR, PLR, SII, LMR and PNI and distant metastasis in GC and develop a practical prediction model for distant metastasis. Subsequently, we will evaluate its reliability and clinical effectiveness.

**Figure 1:**
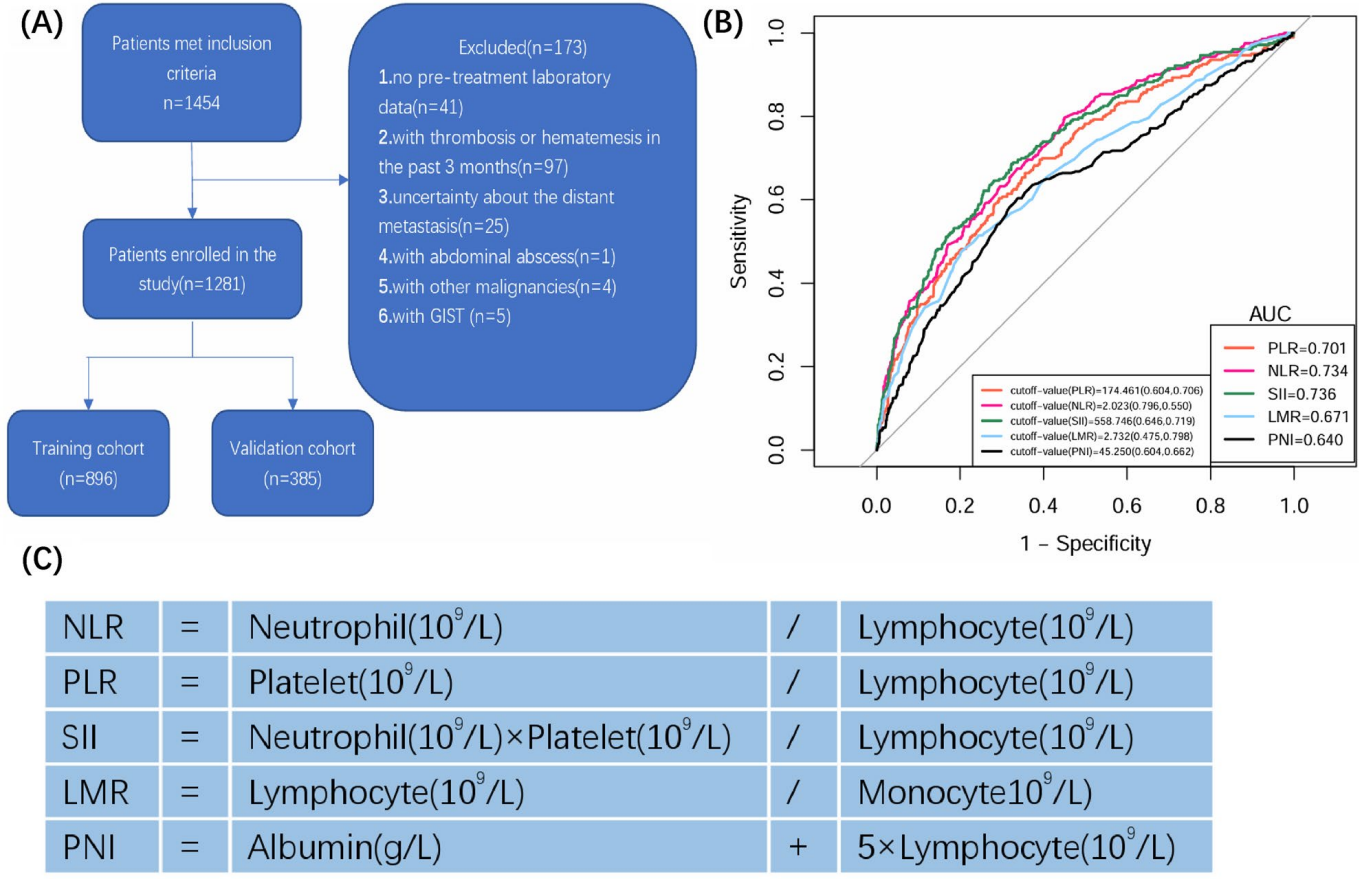
(**A**) Flowchart of patient selection process in the study; (**B**) ROC curves of the composite inflammatory and nutritional markers for predicting distant metastasis in patients with gastric cancer; (**C**) calculation methods for the composite inflammatory and nutritional markers.

## Results

### Patients’ characteristics

A total of 1281 patients were included in our study, with 748 (58.4%) males and 533 (41.6%) females. The median age at the time of diagnosis was 57 years with the age range of 25 to 88 years. 280 (21.9%) patients were diagnosed with distant metastasis. Table 1 summarized the general characteristics of all patients. There were no statistically significant differences in age (p = 0.217) and sex (p = 0.337) between patients with no distant metastasis and distant metastasis. Subsequently, patients were randomly assigned to two cohorts at a ratio of 7:3: consisting of a training cohort with 896 patients and a validation cohort with 385 individuals. As show in Table 2, there were no statistically significant differences in variables between the training cohort and validation cohort, confirming that training and validation sets had similar baseline data.

**Table 1:** Baseline clinical characteristics associated with distant metastasis in gastric cancer patients.

Variable		M1 (n = 280)	M0 (n = 1001)	p-value
Sex	Female	126 (45%)	407 (40.7%)	0.217
	Male	154 (55%)	594 (59.3%)	
Age (years)		$56.1 \pm 11.7$	$56.8\pm 10.9$	0.337
White blood cell count $(10^9/{\text{L}})$		$6.3 \pm 2.3$	$5.4 \pm 1.6$	< 0.001
Red blood cell count $(10^{12}/{\text{L}})$		$4.0 \pm 0.6$	$4.2 \pm 0.6$	< 0.001
Hemoglobin (g/L)	$\ge 120$	125 (44.6%)	647 (64.6%)	< 0.001
	< 120	155 (55.4%)	354 (35.4%)	
Platelet count $(10^9/{\text{L}})$		$258.2 \pm 105.4$	$219.7\pm 73.9$	< 0.001
Neutrophil count $(10^9/{\text{L}})$		$4.3 \pm 2.1$	$3.2 \pm 1.4$	< 0.001
Lymphocyte count $(10^9/{\text{L}})$		$1.3 \pm 0.5$	$1.6\pm 0.6$	< 0.001
Monocyte count $(10^9/{\text{L}})$		$0.5 \pm 0.2$	$0.4\pm 0.2$	< 0.001
Total protein (g/L)	$\ge 60$	208 (74.3%)	835 (83.4%)	< 0.001
	< 60	72 (25.7%)	166 (16.6%)	
Albumin (g/L)	$\ge 35$	210 (75%)	889 (88.8%)	< 0.001
	< 35	70 (25%)	112 (11.2%)	
d-dimer (mg/L)	$\le 0.5$	178 (63.6%)	934 (93.3%)	< 0.001
	> 0.5	102 (36.4%)	67 (6.7%)	
CEA (ng/mL)	${\le 5}$	204 (72.9%)	938 (93.7%)	< 0.001
	> 5	76 (27.1%)	63 (6.3%)	
CA199 (U/mL)	${\le 35}$	177 (63.2%)	908 (90.7%)	< 0.001
	> 35	103 (36.8%)	93 (9.3%)	
CA125 (U/mL)	${\le 35}$	173 (61.8%)	979 (97.8%)	< 0.001
	> 35	107 (38.2%)	22 (2.2%)	
NLR	${\le 2.023}$	57 (20.4%)	551 (55%)	< 0.001
	> 2.023	223 (79.6%)	450 (45%)	
PLR	${\le 174.461}$	111 (39.6%)	707 (70.6%)	< 0.001
	> 174.461	169 (60.4%)	294 (29.4%)	
LMR	${\le 2.732}$	133 (47.5%)	202 (20.2%)	< 0.001
	> 2.732	147 (52.5%)	799 (79.8%)	
SII	${\le 558.746}$	99 (35.4%)	720 (71.9%)	< 0.001
	> 558.746	181 (64.6%)	281 (28.1%)	
PNI	${\le 45.250}$	169 (60.4%)	338 (33.8%)	< 0.001
	> 45.250	111 (39.6%)	663 (66.2%)	
Metastasis site	Peritoneum	111		
	Liver	48		
	Lymph nodes	16		
	Bone	10		
	Ovary	5		
	Lung	2		
	Multiple	88		

M1: patients with distant metastasis; M0: patients without distant metastasis; lymph nods: distant lymph node metastasis beyond regional lymph nodes; multiple: two or more distant metastasis sites.

**Table 2:** Baseline clinical characteristics of patients in training set and validation set and univariate analysis in training set.

Variables		Training cohort	Validation cohort	p	Training cohort		p
		(n=896)	(n=385)		M1(n=198)	M0(n=698)	
Sex	Female	363 (40.5%)	170 (44.2%)	0.250	85 (42.9%)	278 (39.8%)	0.482
	Male	533 (59.5%)	215 (55.8%)		113 (57.1%)	420 (60.2%)	
Age (years)		$56.7 \pm 11.2$	$56.5 \pm 10.8$	0.797	$55.9 \pm 11.4$	$56.9 \pm 11.2$	0.275
White blood cell count $(10^9/{\text{L}})$		$5.6\pm 1.9$	$5.6 \pm 1.8$	0.964	$6.4\pm 2.3$	$5.4 \pm 1.7$	$< 0.001$
Red blood cell count $(10^{12}/{\text{L}})$		$4.1\pm 0.6$	$4.2 \pm 0.6$	0.317	$4.0\pm 0.6$	$4.2 \pm 0.6$	$< 0.001$
Hemoglobin (g/L)	$\ge 120$	535 (59.7%)	237 (61.6%)	0.577	83 (41.9%)	452 (64.8%)	< 0.001
	$< 120$	361 (40.3%)	148 (38.4%)		115 (58.1%)	246 (35.2%)	
Platelet count $(10^9/{\text{L}})$		$228.7\pm 85.9$	$226.7 \pm 77.0$	0.671	$265.3 \pm 111.9$	$218.3 \pm 73.8$	$< 0.001$
Neutrophil count $(10^9/{\text{L}})$		$3.5\pm 1.6$	$3.5 \pm 1.6$	0.939	$4.4\pm 2.1$	$3.2 \pm 1.4$	$< 0.001$
Lymphocyte count $(10^9/{\text{L}})$		$1.5\pm 0.6$	$1.5 \pm 0.5$	0.957	$1.3\pm 0.5$	$1.6 \pm 0.6$	$< 0.001$
Monocyte count $(10^9/{\text{L}})$		$0.4\pm 0.2$	$0.4 \pm 0.2$	0.829	$0.5\pm 0.2$	$0.4 \pm 0.2$	0.002
Total protein (g/L)	$\ge 60$	723 (80.7%)	320 (83.1%)	0.345	152 (76.8%)	571 (81.8%)	0.138
	$< 60$	173 (19.3%)	65 (16.9%)		46 (23.2%)	127 (18.2%)	
Albumin (g/L)	$\ge 35$	760 (84.8%)	339 (88.1%)	0.152	151 (76.3%)	609 (87.2%)	< 0.001
	$< 35$	136 (15.2%)	46 (11.9%)		47 (23.7%)	89 (12.8%)	
d-dimer (mg/L)	$>0.5$	123 (13.7%)	46 (11.9%)	0.440	123 (62.1%)	650 (93.1%)	< 0.001
	$\le 0.5$	773 (86.3%)	339 (88.1%)		75 (37.9%)	48 (6.9%)	
CEA (ng/mL)	${\le 5}$	797 (89%)	345 (89.6%)	0.803	143 (72.2%)	654 (93.7%)	< 0.001
	> 5	99 (11%)	40 (10.4%)		55 (27.8%)	44 (6.3%)	
CA199 (U/mL)	${\le 35}$	761 (84.9%)	324 (84.2%)	0.787	122 (61.6%)	639 (91.5%)	< 0.001
	> 35	135 (15.1%)	61 (15.8%)		76 (38.4%)	59 (8.5%)	
CA125 (U/mL)	${\le 35}$	806 (90%)	346 (89.9%)	1.000	124 (62.6%)	682 (97.7%)	< 0.001
	> 35	90 (10%)	39 (10.1%)		74 (37.4%)	16 (2.3%)	
NLR	${\le 2.023}$	420 (46.9%)	188 (48.8%)	0.561	34 (17.2%)	386 (55.3%)	< 0.001
	> 2.023	476 (53.1%)	197 (51.2%)		164 (82.8%)	312 (44.7%)	
PLR	${\le 174.461}$	578 (64.5%)	240 (62.3%)	0.498	78 (39.4%)	500 (71.6%)	< 0.001
	> 174.461	318 (35.5%)	145 (37.7%)		120 (60.6%)	198 (28.4%)	
LMR	${\le 2.732}$	238 (26.6%)	97 (25.2%)	0.659	98 (49.5%)	140 (20.1%)	< 0.001
	> 2.732	658 (73.4%)	288 (74.8%)		100 (50.5%)	558 (79.9%)	
SII	${\le 558.746}$	572 (63.8%)	247 (64.2%)	0.964	67 (33.8%)	505 (72.3%)	< 0.001
	> 558.746	324 (36.2%)	138 (35.8%)		131 (66.2%)	193 (27.7%)	
PNI	${\le 45.250}$	358 (40%)	149 (38.7%)	0.720	122 (61.6%)	236 (33.8%)	< 0.001
	> 45.250	538 (60%)	236 (61.3%)		76 (38.4%)	462 (66.2%)	
Metastasis	M0	698 (77.9%)	303 (78.7%)	0.807			
	M1	198 (22.1%)	82 (21.3%)				

M1: patients with distant metastasis; M0: patients without distant metastasis.

### Predictive factors selection and construction of nomogram

**Figure 2:**
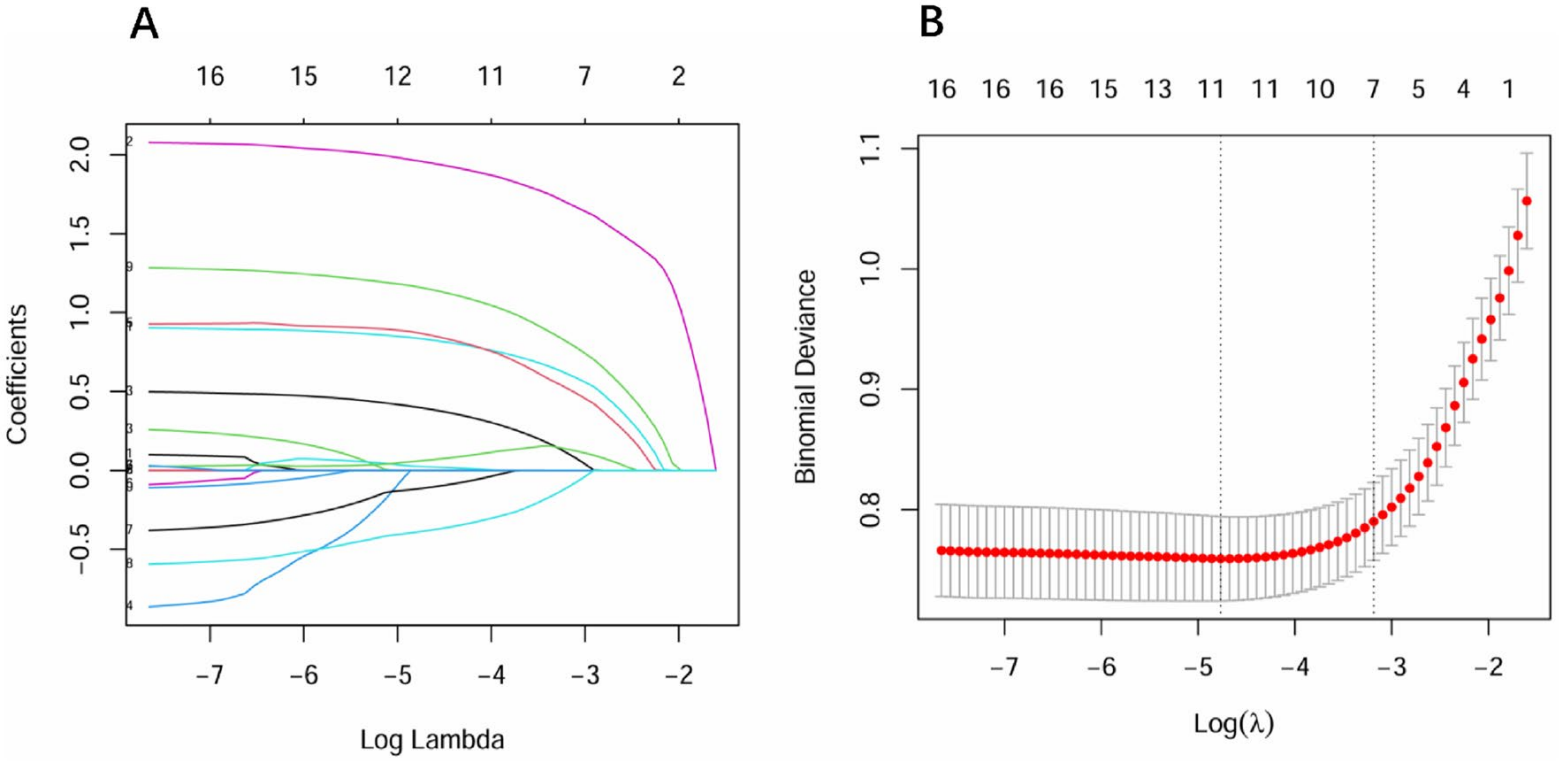
Using Lasso regression to screen potential variables: (**A**) LASSO coefficient profiles of 17 variables; (**B**) Ten-fold cross validation for tuning parameter selection in the LASSO Logistic regression model, the vertical dashed lines represent the optimal values determined by the minimum criteria and 1 − standard error (S.E.) criteria.

**Figure 3:**
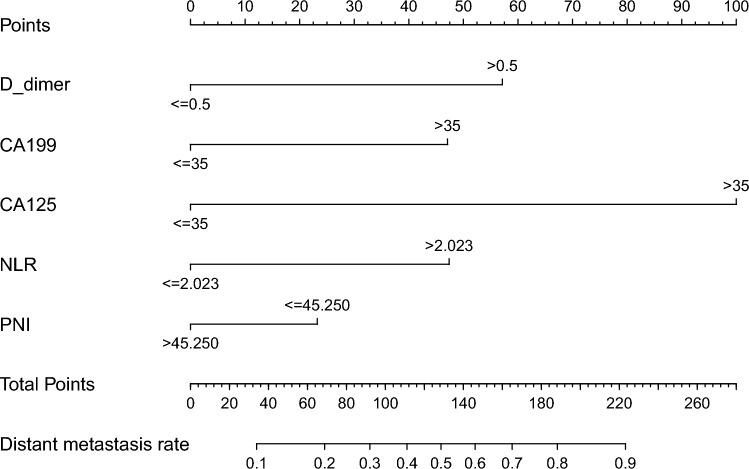
Nomogram for predicting distant metastasis risk in gastric cancer patients.

Univariate analysis of training set in table 2 showed that the variables related to distant metastasis in GC were as follow: White blood cell count (p < 0.001), Red blood cell count (p < 0.001), Hemoglobin (p < 0.001), Platelet count (p < 0.001), Neutrophil count (p < 0.001), Lymphocyte count (p < 0.001), Monocyte count (p = 0.002), Albumin (p < 0.001), d-dimer (p < 0.001), CEA (p < 0.001), CA199 (p < 0.001), CA125 (p < 0.001), NLR (p < 0.001), PLR (p < 0.001), SII (p < 0.001), LMR (p < 0.001) and PNI (p < 0.001). In all 17 associated features (p < 0.05), potential predictors in the training data were selected by Lasso Logistic regression. By selecting lambda.1se, we obtained a model that demonstrated excellent performance with a minimal number of variables. Consequently, seven features with non-zero coefficients, including d-dimer, CA199, CA125, CEA, NLR, SII, and PNI, were selected corresponding to the optimum lambda (lambda.1se = 0.0413174) (Fig 2). Then these variables were evaluated by Multivariate Logistic Regression Analysis (Table 3). As showed in Table 3, d-dimer, CA125, CA199, NLR and PNI were identified as independent predictive factors for predicting distant metastasis in GC. Then we constructed the nomogram based on the above predictors (Fig 3).

**Table 3:** Multivariate Logistic Regression Analysis of distant metastasis in GC patients.

Variables		OR	95%CI	p-value
d-dimer	$>0.5$	3.63	2.18–6.06	$< 0.001$**
	$\le 0.5$	Reference	–	–
CEA	> 5	1.67	0.92–3.01	0.91
	${\le 5}$	Reference	–	–
CA199	> 35	2.55	1.53–4.25	$< 0.001$**
	${\le 35}$	Reference	–	–
CA125	> 35	9.04	4.73–17.26	$< 0.001$**
	${\le 35}$	Reference	–	–
NLR	> 2.023	2.57	1.52–4.33	$< 0.001$**
	${\le 2.023}$	Reference	–	–
SII	> 558.746	1.30	0.80–2.09	0.288
	${\le 558.746}$	Reference	–	–
PNI	> 45.250	0.60	0.40–0.90	0.014*
	${\le 45.250}$	Reference	–	–

OR: odd ratio; CI: confidence interval, *p-value < 0.05, **p-value < 0.001.

### Validation and clinical use of nomogram

**Figure 4:**
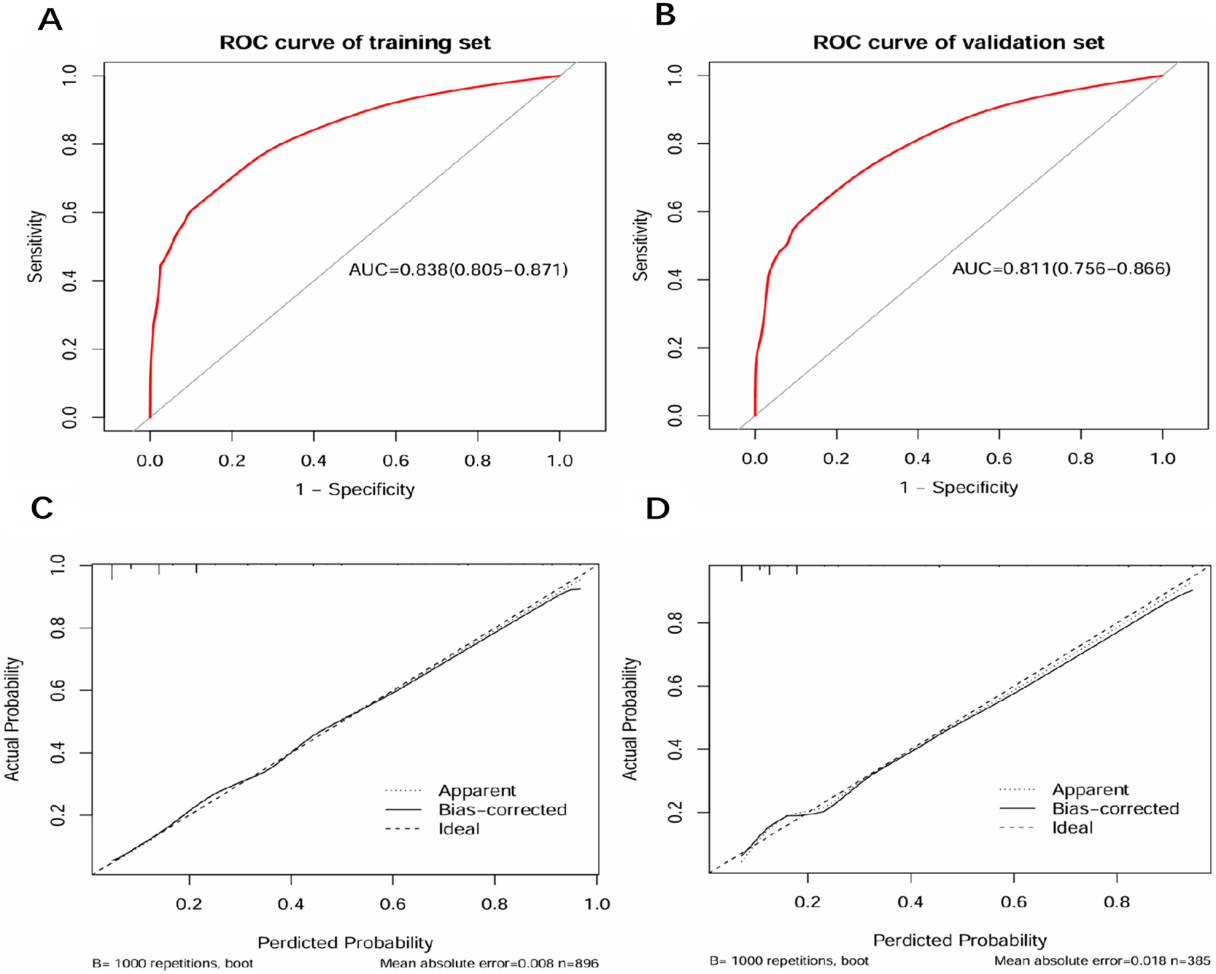
ROC curves and calibration curves of nomogram for predicting distant metastasis in patients with gastric cancer. (**A**) ROC curve of the nomogram in the training cohort; (**B**) ROC curve of the nomogram in the validation cohort; (**C**) the calibration curve of nomogram in the training cohorts; (**D**) the calibration curve of the nomogram in the validation cohorts.

**Figure 5:**
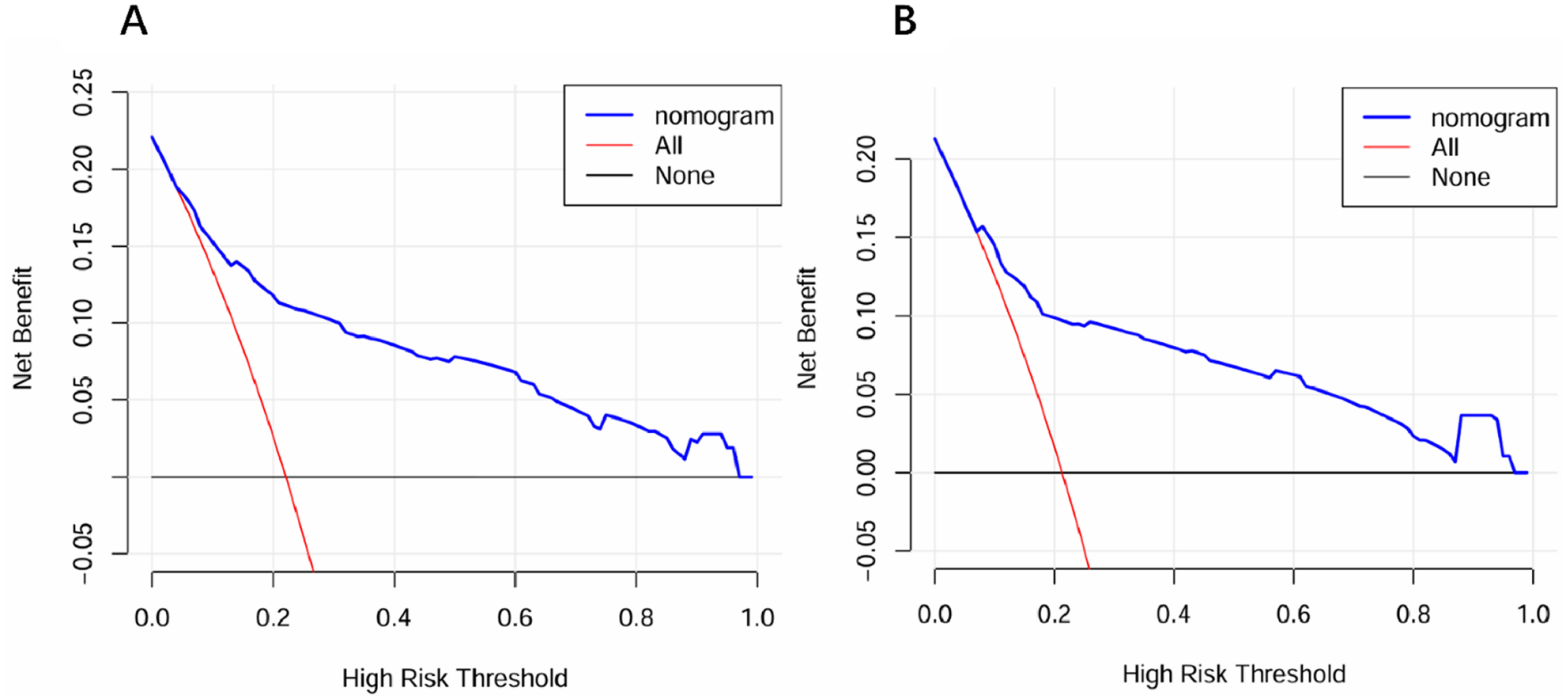
Decision curve analysis of the nomogram for the prediction of distant metastasis in gastric cancer patients. (**A**) Training cohort; (**B**) validation cohort.

The AUC values of our nomogram for predicting distant metastasis were 0.838 in the training set (Fig. 4A) and 0.811 in the validation set (Fig. 4B). The calibration curve of our nomogram for the probability of distant metastasis showed good consistency between prediction and observation in both training (Fig. 4C) and validation cohort (Fig. 4D). Decision curva analysis (DCA) demonstrated that our nomogram conferred a positive net benefit compared to the all-or-none scheme at a threshold probability ranging from 10 to 95% in both training set (Fig. 5A) and testing set (Fig. 5B).

## Discussion

Distant metastasis, the spread of tumor cells from the primary site to distant organs, is often associated with poor prognosis in various cancers. Patients diagnosed with distant metastasis in gastric cancer typically experience significantly lower five-year survival rates compared to those with localized disease^3^. Certainly, the treatment of stomach cancer with distant metastasis is still one of the important challenges faced by clinicians. Accurate prediction of distant metastasis prior to treatment is crucial for patients to avoid unnecessary surgical operations and develop the optimal treatment regimen. PET/CT plays an indispensable role in screening distant metastasis in gastric cancer and has high specificity for detection, but low sensitivity^27^.

The clinical application of liquid biopsy is one of the gastric cancer research hotspots. It has been reported that application of liquid biopsy is feasible for gastric cancer staging. Zeng et al found that folate receptor-positive circulating tumor cells (FR+ CTC ) levels correlated with advanced clinical stage and could effectively predict peritoneal metastasis (PM) in gastric cancer^28^. The data of Pu et al documented that levels of ccf-DNA were elevated in late-stage cancers^29^. Despite demonstrating promising applications, liquid biopsy technology is still in the exploratory phase. The lack of large-scale clinical study validation, standardized operational procedures and data processing methods, and prohibitive costs prevent its widely application in the clinic^30^.

In this study, we explored the association between peripheral blood biomarkers related to inflammation, nutrition, coagulation and tumor markers with distant metastasis in gastric cancer. And we identified that d-dimer, CA125, CA199, NLR and PNI were significantly associated with distant metastasis in GC.

Tumor markers play an important role in predicting the stage of gastric cancer. Nakata et al discovered that CA125 performed better than imaging modalities including computed tomography and ultrasonography in predicting peritoneal dissemination^31^. Li et al found that the positive levels of CA125 in patients with distant metastasis were statistically significant compared to those without distant metastasis and healthy control group while not statistically significant between patients without distant metastasis and healthy control group, which means CA125 related to the distant metastasis of GC^32^. Kochi et al indicated that the positivity rates of CA199 were increased significantly in stage IV than stage III or below (more than 50% vs less than 30%)^33^. These results provide support for our conclusion.

The association between blood coagulation and cancer development is well recognized^34,35^. d-dimer is a soluble fibrin degradation product (FDP) composed of two cross-linked D fragments of the fibrin protein and has been used as a screening and diagnostic tool in numerous coagulopathies and thrombotic disease. In patients with gastric cancer, increasing d-dimer level is associated with advanced clinical pathological stage, more lymph node metastasis, distant metastasis and poor overall survival (OS)^36–38^.

It is estimated that 15–40% cancer patients with malnutrition at diagnosis and 40–80% cases will be malnourished during the treatment of the disease^39^. Malnutrition worsens OS and increase the postoperative complications in cancer patients^40–42^. Cachexia is not an inevitable consequence of cancer, but it is clearly associated with advanced-stage disease^43^. PNI is a simple and effective indictor for assessing nutrition status. Low PNI not only predicts poor survival in cancer patients, but also associated with TNM stage^26,44^.

The chronic and sustained inflammation induced by tumors leads to changes in hematopoiesis and in the systemic composition and functional status of immune cells, thereby promotes metastasis^45^. Studies have reported that systemic inflammation indexes have good predictive and prognostic value in patients with tumors. High levels of NLR were associated with distant metastasis and poor prognosis in gastric cancer^46–49^.

The predictive nomogram based on these factors performed excellent predictive power in both training set and validation. The DCA curves showed that the nomogram had good clinical effectiveness. More significantly, the indicators used in our nomogram were affordable and easily available. Admittedly, there are still some limitations of our study. Firstly, the results of our study require further validation due to the limited sample size and the absence of external data validation. Secondly, the cut-off value of NLR and PNI is still controversial, which means the threshold value in our study may not be applicable to other researches. Thirdly, our research was based on inpatients, the applicability for outpatients needs further exploration. Therefore, further large-scale multicenter prospective studies are necessary to validate the results of our research.

## Conclusion

In conclusion, our study indicated that d-dimer, CA199, CA125, NLR and PNI were independent predictive factors for distant metastasis and developed a nomogram based on these factors. The nomogram performed well in predicting distant metastasis in gastric cancer patients, which means it can be an important screen tool for clinicians and help to provide individualized treatment strategies.

## Methods

### Patients

From January 2018 to April 2023, a total of 1454 inpatients with gastric cancer from Xiangya Hospital were enrolled in this study. Patient’s eligibility criteria for this study are as follows: (1) all patient’s pathology confirmed as adenocarcinoma; (2) no prior treatment with neoadjuvant chemotherapy or surgery before obtaining their first peripheral blood data; (3) not gastric remnant carcinoma. Patients were excluded if they met any of the following criteria: (1) lack of pre-treatment laboratory data; (2) discover thrombosis or hematemesis in the past 3 months (3) uncertainty about the presence or absence of distal spread; (4) with active inflammatory, chronic infection, or autoimmune rheumatic diseases; (5) with other malignancies or gastrointestinal stromal tumor (GIST). The flowchart for the screening process of eligible gastric cancer patients is presented in Fig. 1A. Ultimately, a total of 1281 patients with gastric cancer were screened. 896 patients were assigned to the training cohort, while other 385 patients for validation cohort.

### Data collection and processing

The collection of clinical parameters included basic demographic information (age, sex), hematological parameters (White blood cell count, Red blood cell count, Neutrophil count, Lymphocyte count, Monocyte count, Platelet count, Hemoglobin, Total protein, Albumin, d-dimer), and tumor markers (CEA, CA125, CA199). All laboratory blood test data were collected from tests performed on the patients’ first admission prior to any treatment. Distant metastasis was classified according to the 8th AJCC tumor classification and obtained from the hospital medical records. And five composite inflammatory and nutritional markers (NLR, PLR, SII, LMR and PNI) were obtained from hematological indexes. Figure 1C presented the calculation method of each composite indexes. The established upper normal limits for CEA, CA199 and CA125, were 5 ng/mL, 35 U/mL and 35 U/mL. Receiver operating curve (ROC) was used to determine the optimal cut-off values for composite inflammatory and nutritional markers by calculating the maximal Youden index as shown in Fig. 1B. Tumor markers and composite markers were divided into two groups based on their thresholds or cutoff values. Hypoalbuminemia was defined as Albumin < 35 g/L. Total protein and d-dimer levels were divided into two groups based on reference values, and Albumin divided into two groups based on the presence or absence of hypoproteinemia.

### Development and validation of the nomogram

Lasso regression and multivariate Logistic regression were used to select independent predictive factors from the training cohort. Subsequently, a nomogram was constructed using the independent factors identified through multivariate analysis. Area under the ROC curves (AUC) and calibration curves were utilized to assess the predictive ability of the nomogram. Additionally, the decision curve analysis (DCA) was used to evaluated the clinical utility of the nomogram by quantifying the net benefits.

### Statistical analysis

The statistical analysis was performed with R studio (version 4.3.1). Continuous variables were presented as mean and standard deviation, and categorical variables were presented as numbers and percentages. The p-value < 0.05 was considered statistically significant. Univariate analysis in Tables 1 and 2 were performed by the “autoReg” package. The “glmnet” package was utilized to perform Lasso binary logistic regression. And the “rms” package was employed to perform multivariate binary logistic regression, visualization of nomogram and plot calibration curve. ROC curves for assessing the discriminatory power of the nomogram and identifying the optimal cut-off values was done with pROC package. And decision curve analysis was performed with the “rmda” package.

### Ethical approval and consent to participate

The retrospective design of the study received approval from the Ethics Committee of Xiangya Hospital (approval no. 20200237). The procedures used in this study adhere to the tenets of the Declaration of Helsinki. As this was a retrospective observational study, informed consent was waived by the Ethics Committee of Xiangya Hospital.

## Data Availability

The dataset utilized and analyzed in the current research is accessible from the corresponding authors upon reasonable request.

## References

[1] Sung H (2021). Global cancer statistics 2020: Globocan estimates of incidence and mortality worldwide for 36 cancers in 185 countries. CA Cancer J. Clin..

[2] Kakeji Y (2022). A retrospective 5-year survival analysis of surgically resected gastric cancer cases from the Japanese gastric cancer association nationwide registry (2001–2013). Gastric Cancer.

[3] Thrift AP, El-Serag HB (2020). Burden of gastric cancer. Clin. Gastroenterol. Hepatol..

[4] Ajani JA (2022). Gastric cancer, version2.2022, NCCN clinical practice guidelines in oncology. J. Natl. Compr. Cancer Netw..

[5] Lordick F (2022). Gastric cancer: ESMO clinical practice guideline for diagnosis, treatment and follow-up. Ann. Oncol..

[6] Wang F-H (2021). The Chinese Society of Clinical Oncology (CSCO): Clinical guidelines for the diagnosis and treatment of gastric cancer, 2021. Cancer Commun..

[7] Diakos CI, Charles KA, McMillan DC, Clarke SJ (2014). Cancer-related inflammation and treatment effectiveness. Lancet Oncol..

[8] Grivennikov SI, Greten FR, Karin M (2010). Immunity, inflammation, and cancer. Cell.

[9] Maiorino L, Daßler-Plenker J, Sun L, Egeblad M (2022). Innate immunity and cancer pathophysiology. Annu. Rev. Pathol..

[10] Hibino S (2021). Inflammation-induced tumorigenesis and metastasis. Int. J. Mol. Sci..

[11] de Martel C, Georges D, Bray F, Ferlay J, Clifford GM (2020). Global burden of cancer attributable to infections in 2018: A worldwide incidence analysis. Lancet Glob. Health.

[12] Chen J, Hong D, Zhai Y, Shen P (2015). Meta-analysis of associations between neutrophil-to-lymphocyte ratio and prognosis of gastric cancer. World J. Surg. Oncol..

[13] Zhang X (2020). Clinicopathological and prognostic significance of platelet-lymphocyte ratio (PLR) in gastric cancer: An updated meta-analysis. World J. Surg. Oncol..

[14] Zhang L-X, Wei Z-J, Xu A-M, Zang JH (2018). Can the neutrophil-lymphocyte ratio and platelet-lymphocyte ratio be beneficial in predicting lymph node metastasis and promising prognostic markers of gastric cancer patients? Tumor maker retrospective study. Int. J. Surg..

[15] Ma JY, Liu Q (2018). Clinicopathological and prognostic significance of lymphocyte to monocyte ratio in patients with gastric cancer: A meta-analysis. Int. J. Surg..

[16] Zhang J (2023). Single and combined use of the platelet-lymphocyte ratio, neutrophil-lymphocyte ratio, and systemic immune-inflammation index in gastric cancer diagnosis. Front. Oncol..

[17] Templeton AJ, Mcnamara MG, Seruga B, Vera-Badillo FE, Amir E (2014). Prognostic role of neutrophil-to-lymphocyte ratio in solid tumors: A systematic review and meta-analysis. JNCI J. Natl. Cancer Inst..

[18] Ethier J-L, Desautels D, Templeton A, Shah PS, Amir E (2017). Prognostic role of neutrophil-to-lymphocyte ratio in breast cancer: A systematic review and meta-analysis. Breast Cancer Res..

[19] Shui Y (2021). Prognostic and clinicopathological significance of systemic immune-inflammation index in pancreatic cancer: A meta-analysis of 2,365 patients. Aging.

[20] Muscaritoli M (2017). Prevalence of malnutrition in patients at first medical oncology visit: The PreMiO study. Oncotarget.

[21] Yang Y (2016). The prognostic nutritional index is a predictive indicator of prognosis and postoperative complications in gastric cancer: A meta-analysis. Eur. J. Surg. Oncol..

[22] Nogueiro J (2022). The impact of the prognostic nutritional index (PNI) in gastric cancer. Langenbecks Arch. Surg..

[23] Xue Y, Zhou X, Xue L, Zhou R, Luo J (2019). The role of pretreatment prognostic nutritional index in esophageal cancer: A meta-analysis. J. Cell. Physiol..

[24] Li JH (2023). Preoperative albumin-to-globulin ratio and prognostic nutritional index predict the prognosis of colorectal cancer: A retrospective study. Sci. Rep..

[25] Niu Z, Yan B (2023). Prognostic and clinicopathological effect of the prognostic nutritional index (PNI) in patients with cervical cancer: A meta-analysis. Ann. Med..

[26] Dai M, Sun Q (2023). Prognostic and clinicopathological significance of prognostic nutritional index (PNI) in patients with oral cancer: A meta-analysis. Aging.

[27] Lehmann K (2017). 18FDG-PET-CT improves specificity of preoperative lymph-node staging in patients with intestinal but not diffuse-type esophagogastric adenocarcinoma. Eur. J. Surg. Oncol..

[28] Zeng CDD (2022). Preoperative folate receptor-positive circulating tumor cells are associated with occult peritoneal metastasis and early recurrence in gastric cancer patients: A prospective cohort study. Front. Oncol..

[29] Pu WY (2016). Prediction of cancer progression in a group of 73 gastric cancer patients by circulating cell-free DNA. BMC Cancer.

[30] Ma S (2023). Clinical application and detection techniques of liquid biopsy in gastric cancer. Mol. Cancer.

[31] Nakata B (1998). Serum CA 125 level as a predictor of peritoneal dissemination in patients with gastric carcinoma. Cancer Interdiscip. Int. J. Am. Cancer Soc..

[32] Li X, Li S, Zhang Z, Huang D (2022). Association of multiple tumor markers with newly diagnosed gastric cancer patients: A retrospective study. PeerJ.

[33] Kochi M (2000). Evaluation of serum CEA and CA19-9 levels as prognostic factors in patients with gastric cancer. Gastric Cancer.

[34] Wojtukiewicz MZ, Hempel D, Sierko E, Tucker SC, Honn KV (2016). Thrombin-unique coagulation system protein with multifaceted impacts on cancer and metastasis. Cancer Metastasis Rev..

[35] Falanga A, Marchetti M, Vignoli A (2013). Coagulation and cancer: Biological and clinical aspects. J. Thromb. Haemost..

[36] Zhang X (2022). d-dimer, a predictor of bad outcome in gastric cancer patients undergoing radical resection. Sci. Rep..

[37] Dongmei D (2014). d-dimer: Not just an indicator of venous thrombosis but a predictor of asymptomatic hematogenous metastasis in gastric cancer patients. PLoS ONE.

[38] Liu L (2014). Elevated plasma d-dimer levels correlate with long term survival of gastric cancer patients. PLoS ONE.

[39] Ravasco P (2019). Nutrition in cancer patients. J. Clin. Med..

[40] Matsui R, Rifu K, Watanabe J, Inaki N, Fukunaga T (2023). Impact of malnutrition as defined by the glim criteria on treatment outcomes in patients with cancer: A systematic review and meta-analysis. Clin. Nutr..

[41] Zheng H-L (2017). Effects of preoperative malnutrition on short- and long-term outcomes of patients with gastric cancer: Can we do better?. Ann. Surg. Oncol..

[42] Morgan TM (2011). Preoperative nutritional status is an important predictor of survival in patients undergoing surgery for renal cell carcinoma. Eur. Urol..

[43] Baracos VE, Martin L, Korc M, Guttridge DC, Fearon KC (2018). Cancer-associated cachexia. Nat. Rev. Dis. Primers.

[44] Sun K, Chen S, Xu J, Li G, He Y (2014). The prognostic significance of the prognostic nutritional index in cancer: A systematic review and meta-analysis. J. Cancer Res. Clin. Oncol..

[45] Garner H, de Visser KE (2020). Immune crosstalk in cancer progression and metastatic spread: A complex conversation. Nat. Rev. Immunol..

[46] Nakayama Y (2014). Usefulness of the neutrophil/lymphocyte ratio measured preoperatively as a predictor of peritoneal metastasis in patients with advanced gastric cancer. Surg. Today.

[47] Kim EY, Song KY (2020). The preoperative and the postoperative neutrophil-to-lymphocyte ratios both predict prognosis in gastric cancer patients. World J. Surg. Oncol..

[48] Wang H (2020). Prognostic value of neutrophil-lymphocyte ratio, platelet-lymphocyte ratio, and combined neutrophil-lymphocyte ratio and platelet-lymphocyte ratio in stage iv advanced gastric cancer. Front. Oncol..

[49] Zhang X (2022). Predictive value of neutrophil-to-lymphocyte ratio for distant metastasis in gastric cancer patients. Sci. Rep..

